# Behavioural flexibility in spider mites: oviposition site shifts based on past and present stimuli from conspecifics and predators

**DOI:** 10.1098/rsos.170328

**Published:** 2017-07-19

**Authors:** Aoi Murase, Kazuo Fujita, Shuichi Yano

**Affiliations:** 1 Laboratory of Ecological Information, Graduate School of Agriculture, Kyoto University, Kyoto, Japan; 2 Department of Psychology, Graduate School of Letters, Kyoto University, Kyoto, Japan

**Keywords:** Acari, predation avoidance, oviposition site shift, invertebrate learning

## Abstract

Predator-experienced individuals often change their predation avoidance response when they re-encounter the same predators or their cues. Recent reports show that behavioural change sometimes occurs even before the re-encounter. To function as an adaptive strategy in the wild, such prospective experience-induced behaviour should change flexibly in response to changing situations. We assessed flexibility of experience-induced oviposition site shift in two closely related species of spider mites, *Tetranychus kanzawai* and *T. urticae*, from the viewpoint of reducing future predation risk on their eggs. We found that: (i) individuals of *T. kanzawai* shifted oviposition site depending on the presence of conspecific eggs; (ii) after experiencing predation threat *T. kanzawai* females shifted oviposition site even in the absence of any current predation threat; (iii) this experience-induced shift of oviposition site was weakened in the presence of conspecific males; and (iv) experience-induced behaviour was retained for a shorter period in *T. urticae* than in *T. kanzawai*, possibly because the demand for learning may differ with regard to biological conditions encountered in the wild.

## 1. Introduction

Experience of predators changes various aspects of anti-predator behaviours in many animal species. For example, larvae of the fruit fly *Drosophila melanogaster* avoid odours associated with predation more actively if they have experienced threat of predation [1]. Predator-experienced Coho salmon (*Oncorhynchus kisutch*) also show stronger predator avoidance, resulting in longer survival than naive salmon in the presence of predators [2]. Similar enhanced predator avoidance based on experience has been observed in hare-wallabies (*Lagorchestes hirsutus*) [3] and freshwater snails [4]. These studies show sensitization to stimuli from predators. By contrast, some experienced animals habituate to predation threat, i.e. they respond less actively to predators. For example, predator-experienced spider mites attenuated their anti-predator responses to predator cues [5].

Although several studies have focused on effects of prior predator experience during re-encounters with predation threat, recent studies have investigated behavioural change in the absence of current threat. Hirayama & Kasuya [6] reported that water striders *Aquarius paludum insularis* changed oviposition depth in response to an encounter with a parasitoid wasp, and that the change became weaker over time. *Drosophila melanogaster* changed their preferred oviposition substrate for 2 days following a single exposure to parasitoid wasps [7]. These studies reveal different processes than simple sensitization or habituation in arthropods, but the precise nature of these processes and its adaptability is unclear. As mentioned by Menzel [8], to understand the adaptability of learning, more attention should be paid to the influence of events following the experience that induced behavioural change (e.g. influence of cohabitation with conspecifics after predator experience-induced ovipositional change). Bleeker *et al*. [9] proposed a relationship between learning functions and ecological traits. They showed that two closely related parasitoid species, *Cotesia glomerata* and *C. rubecula*—the former gregarious and the latter solitary—show different retention periods of odour preference induced by oviposition on a caterpillar. Comparing the effects of experience between two closely related species with different ecological characteristics would also provide circumstantial evidence of adaptive learning. In this study, we examined whether learned predation risks and current interactions affect oviposition behaviours of two closely related phytophagous mites, *Tetranychus kanzawai* and *T. urticae*.

**Figure 1:**
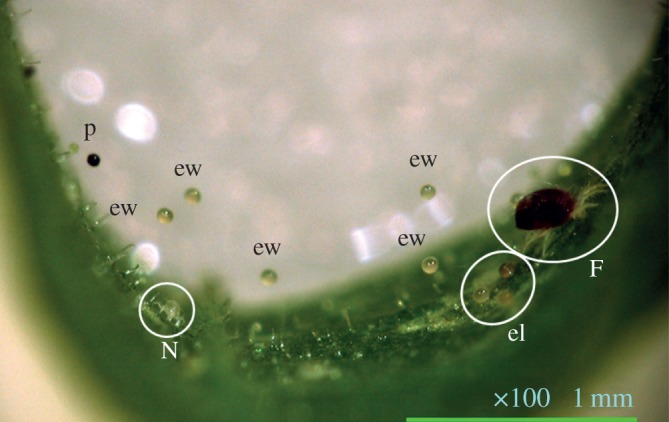
The oviposition site of female *T. kanzawai* in the presence of the predatory mite *N. womersleyi.* ew: eggs laid on webs, el: eggs laid on the leaf surface, p: pellet (faeces), F: *T. kanzawai* female, N: larva of *N. womersleyi*. The green bar (right bottom) represents 1 mm.

Spider mites of the genus *Tetranychus*, major leaf-sapping pests all over the world, are well-studied experimental animals. Our main subjects were *T. kanzawai*, commonly found on wild plants in Japan. They construct intricate three-dimensional webs on leaves, in partial contact with the leaf surface [10]. Females of this mite lay eggs usually on the leaf surface, and on the webs only in the presence of predatory mites [11–14] (figure 1), which implies the behaviour incurs fitness cost to the mites. Eggs laid on the webs are predated less frequently than those on the leaf surface [13,14]. On the other hand, although eggs laid on the leaf surface are more likely to be preyed upon than those laid on the webs, the difference would be mitigated by the presence of conspecific eggs through dilution effect [15] since the mites often aggregate on their host plant [16]. Therefore, we hypothesize that oviposition site shift of *T. kanzawai* females from the leaf surface onto the webs should be promoted by learned predation risks, while reduced by indicators of conspecific eggs. Therefore, we first examined whether the presence of conspecific eggs leads to conservative oviposition on the leaf surface in naive *T. kanzawai* (Experiment 1). Second, we predicted that predation-threat experience in *T. kanzawai* would induce oviposition site shift from the leaf surface onto the webs even in the absence of current predation threat (Experiment 2). We next examined whether the presence of conspecifics would affect the oviposition behaviour of predation threat-experienced mites (Experiment 3). Because *T. kanzawai* mites often aggregate on their host plant [16] and mated females are considered the dispersal stage of the mite [17], adult males remain in their natal colony as a rule, and thus tend to co-occur with conspecific eggs. Moreover, copulatory attempts (i.e. physical contacts) made by conspecific males on predation threat-experienced females would be a detectable indicator of conspecific eggs. Our final question was whether the retention period of predator experience-induced change in oviposition differs between two closely related species, *T. kanzawai* and *T. urticae*. *T. urticae* mites spin silk thread [18] and construct three-dimensional webs on leaves similarly to *T. kanzawais* [10]. Both *T. urticae* and *T. kanzawai* are polyphagous spider mites [19,20]; they live together on many wild and cultivated host plants and often co-occur on the same plant specimen [17,21,22]. *Tetranychus kanzawai* lives on wild plants where predators are abundant, whereas *T. urticae* in Japan is observed only in agro-ecosystems where predators are less abundant because pesticides kill not only pest mites but also predatory mites (i.e. *T. kanzawai* potentially encounters predators more frequently than *T. urticae* does) [17]. Due to the biological differences between these two species, their learning requirements in the context of predation avoidance may be different. We therefore examined if predation-experienced *T. urticae* oviposits more eggs on the web in the absence of predators in the same way as *T. kanzawai* (Experiment 4)*.*

## 2. Methods

### 2.1. Mites and plants

The *T. kanzawai* study population was collected from kudzu vine (*Pueraria lobate*) in Kyoto, Japan, in 2014, and maintained on an expanded primary leaf (the first seedling leaf) of common bean, *Phaseolus vulgaris*. The leaf was pressed onto water-saturated cotton in a Petri dish (90 mm in diameter, 14 mm in depth), and we made this leaf dish for mites 5 to 7 time per week. The age of newly emerged female mites was estimated based on the day each dish was started by introducing 10 females and 2 males. *Phaseolus vulgaris* was reared in a thermostatic room at 25°C and 65% RH, with a photoperiod of 16 h : 8 h (L : D). The *T. urticae* study population was collected from strawberry (*Fragaria chiloensis var. ananassa*) plants in Shizuoka, Japan, in 2013 and were reared in the same manner. No mites of *T. kanzawai* and *T. urticae* had previous experience of predation threats.

Individuals of a specialist predatory mite *Neoseiulus womersleyi* were used in our experiments. These are a common natural enemy of tetranychid mites that can penetrate their prey species' webs [23], but eat more eggs on the leaf surface than on webs [12–14]. *Neoseiulus womersleyi* were collected in Nara, Japan, in 2010, and were reared on bean leaf dishes heavily infested with the prey species *T. urticae* (30–50 adult and immature females per leaf). The population of *T. urticae* used for rearing *N. womersleyi* was different from the population used in the following experiment. All dishes were placed in a transparent plastic container and kept at 25°C and 65% RH, with a photoperiod of 16 h : 8 h (L : D) throughout the study.

**Figure 2.**
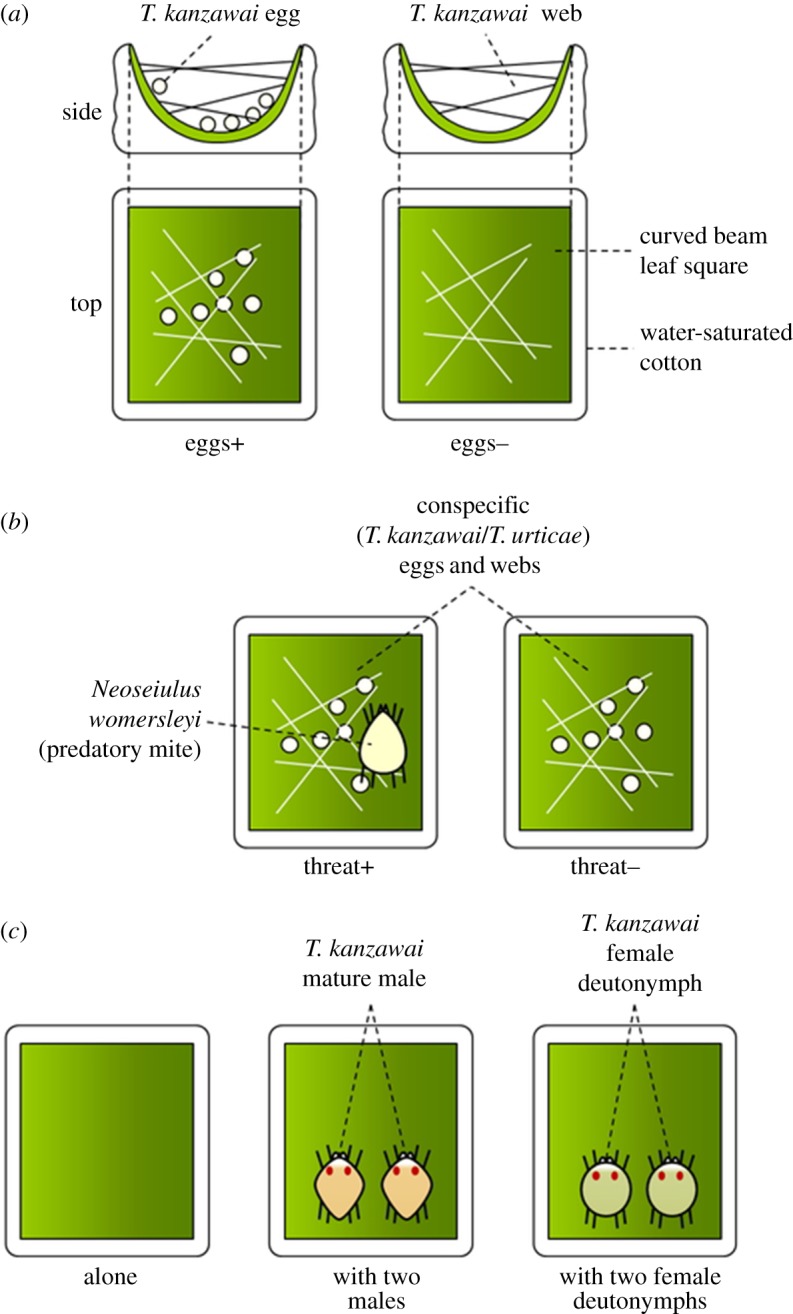
Experimental set-ups. (*a*) In Exp. 1 females of *T. kanzawai* cohabited with conspecific eggs and webs (‘eggs+’) or webs alone (‘eggs−’) for 3 days. (*b*) The threat+ *T. kanzawai* females (Exp. 2) and *T. urticae* females (Exp. 4) cohabited with both a predator and conspecific eggs, whereas threat− females lived only with conspecific eggs. (*c*) After exposure to a predatory mite, each female was transferred to a new curved leaf square having either no conspecifics (‘alone’), two mature conspecific males (‘with two males’) or two female deutonymphs.

All procedures in the following Exps. 1 to 4 took place on a 1 × 1 cm leaf square of *Phaseolus vulgaris* on water-saturated cotton in plastic Petri dishes. Each leaf square was curved in a semi-cylindrical shape to prevent further leaf deformation by *T. kanzawai* females [12] and to standardize the patch structure for building three-dimensional webs on the leaves. The water around the leaf square prevented mites from dispersing during the experiments. The set-ups for each experiment are described separately below and illustrated in figure 2.

### 2.2. Experiment 1: Oviposition site shift when cohabiting with conspecific eggs

We prepared curved bean leaf squares with conspecific webs and eggs, or with webs alone. First, we introduced three *T. kanzawai* females 2–4 days or 1.5 h after maturation (hereafter ‘2–4 days females’ and ‘1.5 h females’), and let them construct webs and oviposit for 20 h. To prepare 1.5 h females, teleiochrysalis phase females were selected randomly from the population containing hundreds of such individuals. The selected females were transferred together to a new leaf on a Petri dish, and matured simultaneously by controlling relative humidity (for details, see [24]). Then, an equal number of *T. kanzawai* males were introduced onto the leaf, and the females and males remained together to mate for 1.5 h. *Tetranychus kanzawai* females do not lay eggs within 24 h after maturation under laboratory conditions [25], so 1.5 h females constructed webs with no eggs, whereas 2–4 days females laid eggs on the leaf square. Next, we removed females from each leaf square with minimal disturbance, and introduced a randomly selected teleiochrysalis female and two matured males onto both leaf squares with eggs and webs (hereafter ‘eggs+’) and with webs alone (‘eggs−’). After 1 day, the females were matured simultaneously by controlling relative humidity. Each female spent 2 h with two males and were allowed to mate. After we removed males, all leaf squares were kept in transparent plastic containers at 25°C and 65% RH, with a photoperiod of 16 h : 8 h (L : D). After 3 days, the number of eggs laid on the web and on the leaf surface was counted separately. For eggs+, egg numbers were calculated by subtracting the initial egg numbers from the cumulative egg numbers. Total number of eggs, which may reflect possible effects caused by preceding females, was also compared between the treatments. The number of replicates for each treatment pair (eggs+ : eggs−) was 12 : 10.

### 2.3. Experiment 2: Ovipositional change after threat experience

First, three 2–4 days *T. kanzawai* females were introduced onto a curved bean leaf square for 24 h to let them lay sufficient numbers of eggs (*n* > 20), and then removed with minimal disturbance. Second, 1.5 h *T. kanzawai* mated females, prepared in the same manner as in Exp. 1, were separately introduced onto egg-laid leaf squares. An adult female *N. womersleyi*, the predator, was then immediately introduced onto half of the leaf squares (hereafter referred to as ‘threat+’), while the remaining leaf squares served as controls (hereafter ‘threat−’). Therefore, threat+ females lived with both a predator and conspecific eggs, whereas threat− females lived only with conspecific eggs. *Neoseiulus womersleyi* prefers eggs to adult females [25]; the latter are seldom preyed upon if eggs are available. We kept the leaf squares at 25°C and 65% RH for 19 h, from 14.30 to 09.30 the following day. As mentioned above, *T. kanzawai* females would not have started oviposition during this 19 h period [25]. Within this period, the leaf squares were in darkness from 23.00 to 07.00. At 09.30, we transferred threat+ and threat− females onto new leaf squares with no trace of conspecifics or predators. Starting 24 h after this transfer, the number of eggs laid on the web and on the leaf surface was counted separately for 4 days. Total number of eggs, which may reflect non-lethal effects of predators [26], was also compared between the treatments. The number of replicates for each treatment pair (threat+ : threat−) was 12 : 11. Only on day 1, one female in threat− did not lay eggs. This female was excluded from the analysis of proportion of eggs on the web, but included in the analysis of number of eggs on day 1.

### 2.4. Experiment 3: Effect of cohabitation with conspecifics after threat experience

In the same manner as in Exp. 2, each 1.5 h female was exposed to a predator. Following the exposure, each female was transferred onto a new leaf square having either no conspecifics (‘alone’), two mature conspecific males (‘with two males’) or two female deutonymphs, whose body size was similar to a mature male (‘with two female deutonymphs’). The conspecific individuals were randomly selected from the study population. After 24 h, we counted numbers of eggs on the web and on the leaf surface as described above. The number of replicates for each treatment (alone: with two males: with two female deutonymphs) was 20 : 23 : 21.

### 2.5. Experiment 4: Ovipositional change by *T. urticae* females induced by threat experience

As in Exp. 2, each 1.5 h *T. urticae* female was exposed to a predator on a leaf square with eggs laid by 2–4 days *T. urticae*. After keeping the leaf squares at 25°C and 65% RH for 19 h, from 14.30 to 09.30 the following day (23.00 to 07.00, in darkness), we transferred both threat+ and threat− females onto new curved bean leaf squares that had no traces of conspecifics or predators. Starting 24 h after this transfer, the numbers of eggs laid on the web and on the leaf surface were separately counted for 4 days. Total number of eggs, which may reflect possible disturbances caused by conspecific males, was also compared between the treatments. The number of replicates for each treatment pair (threat+ : threat−) was 17 : 10.

### 2.6. Data analysis

In Exps. 1, 2 and 4, the ratio of eggs laid on the webs and total number of eggs were analysed by generalized linear model (hereafter GLM). In Exp. 3 the mean of all pairs were compared using the non-parametric Steel-Dwass test, because the data did not fit with specific probability distribution. All statistical analyses were performed using JMP 9 (SAS Institute, 2010).

## 3. Results

### 3.1. Experiment 1: Oviposition site shift when cohabiting with conspecific eggs

**Figure 3.**
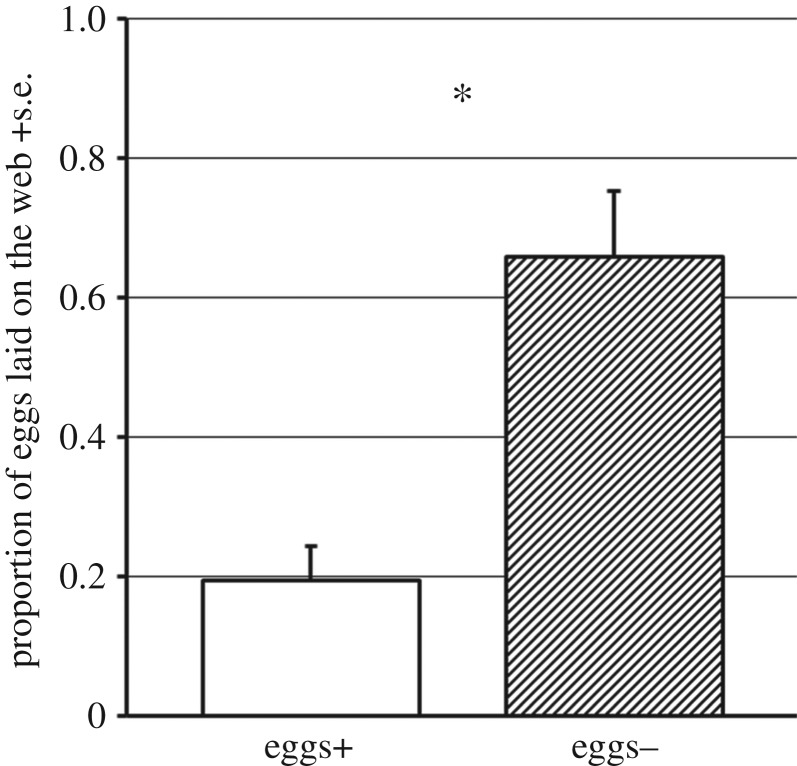
The effect of presence of conspecific eggs on oviposition behaviour of *T. kanzawai* females. The proportion of eggs laid on the web by eggs+ and eggs− females (*p < 0.05; GLM, binomial).

The number of conspecific eggs laid on each leaf square was 12.17 ± 1.12. The proportion of conspecific eggs laid on the web was 0.081 ± 0.030. Females in the presence of conspecific eggs (eggs+) on average laid significantly fewer eggs on the web than females with no eggs present (eggs−) (*p < 0.05; GLM, binomial) (figure 3). The total number of eggs per female after 3 days was not significantly different between eggs+ and eggs− (*p* = 0.625; GLM, Poisson).

### 3.2. Experiment 2: Ovipositional change after threat experience

**Figure 4.**
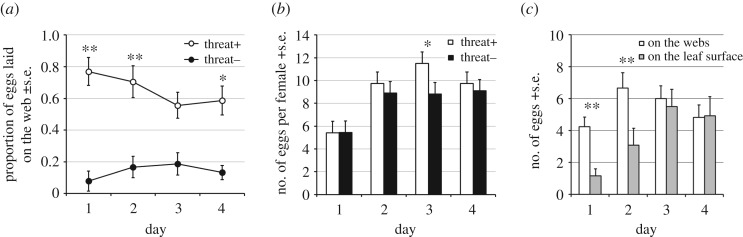
The effect of threat experience on oviposition behaviour of *T. kanzawai* females. (*a*) The proportion of eggs laid on the web by threat+ and threat− females (*p < 0.05, **p < 0.01; GLM, binominal). (*b*) The number of eggs per female per day for threat+ and threat− females (*p < 0.05; GLM, Poisson). (*c*) The number of eggs laid on webs and on the leaf surface by threat+ females (**p < 0.01; GLM, Poisson).

Predator-experienced (threat+) females on average laid a higher proportion of their eggs on the web compared with non-experienced (threat−) females on days 1, 2 and 4 (*p < 0.05, **p < 0.01; GLM, binomial) (figure 4*a*). The number of eggs per female per day (hereafter eggs/female/day) of threat+ young females was significantly higher on day 3 compared to threat− (*p < 0.05; GLM, Poisson) (figure 4*b*), whereas the total number of eggs laid over 4 days per female (hereafter eggs/female/4 days) was not significantly different (*p* = 0.091; GLM, Poisson); threat+ : control = 36.42 ± 2.19 : 32.27 ± 3.02. On days 1 and 2, threat+ females laid significantly more eggs on webs than on leaf surface (**p < 0.01; GLM, Poisson) (figure 4*c*).

### 3.3. Experiment 3: Effect of cohabitation with conspecifics after threat experience

**Figure 5.**
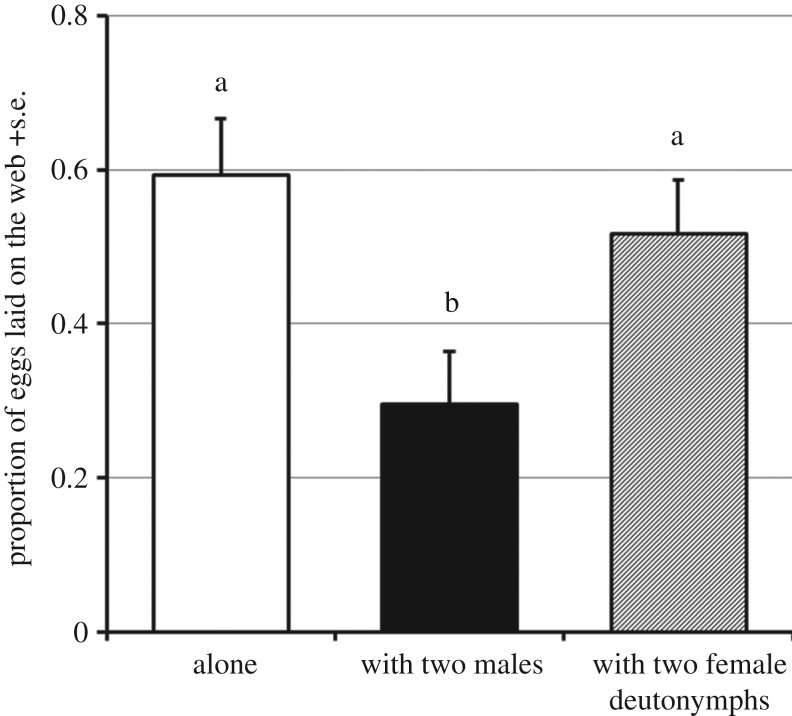
The effect of the conspecific presence on oviposition site shift by threat-experienced *T. kanzawai* females*.* The proportion of eggs laid on the web. Different letters indicate significant differences at *p* < 0.05 (Steel-Dwass all pairs).

Threat-experienced young females laid a lower proportion of their eggs on the web when they lived together with males compared with solitary females and those living together with deutonymphs (figure 5). The number of eggs/female/day was not significantly affected by the presence of conspecifics (Steel-Dwass all pairs).

### 3.4. Experiment 4: Ovipositional change by *T. urticae* females induced by threat experience

**Figure 6.**
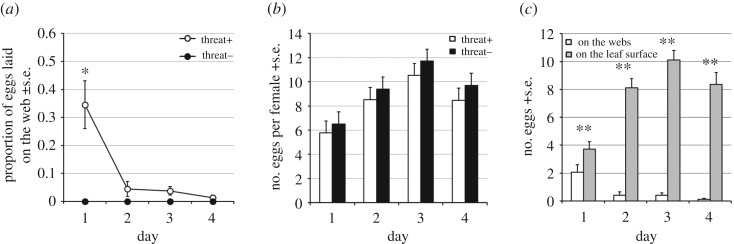
The effect of threat experience on oviposition behaviour of *T. urticae* females. (*a*) The proportion of eggs laid on the web by threat+ and threat− females (*p < 0.05; GLM, binomial). (*b*) The number of eggs per female per day did not differ significantly between threat+ and threat− females on any day (GLM, Poisson). (*c*) The number of eggs on the webs and on the leaf surface by threat+ females (**p < 0.01; GLM, Poisson).

In the absence of a predator experience, *T. urticae* females laid almost all of their eggs on the leaf (figure 6*a*). Predator-experienced (threat+) females on average laid a higher proportion of their eggs on the web compared with non-experienced (threat−) females only on day 1 (*p < 0.05; GLM, binomial) (figure 6*a*). The number of eggs/female/day did not differ significantly between threat+ and threat− females on any day (GLM, Poisson) (figure 6*b*). The total number of eggs/female laid over 4 days was not significantly different between the treatments (*p* = 0.0885; GLM; threat+ : threat− was 33.29 ± 1.98 : 37.30 ± 1.79). The threat+ females laid significantly more eggs on the leaf surface than on webs through day 1 to 4 (**p < 0.01; GLM, Poisson) (figure 6*c*).

## 4. Discussion

We have demonstrated that both past experience and current interactions affect oviposition site selection in *Tetranychus* mites. That is, oviposition site of the mites is affected by learned predation risks and the presence of conspecific eggs.

Experiment 1 showed that *T. kanzawai* females laid more eggs on webs in the absence of conspecific eggs (figure 3). Although the oviposition site shift from leaf surface to web in *T. kanzawai* had been interpreted as a unique response to current predation risk [11,12], our result shows that oviposition on the web can happen in solitude without any such exposure. Presence of conspecific eggs is likely to be a stimulus to change oviposition site from webs to the leaf surface. Almost all conspecific eggs were laid on the leaf surface, so laying eggs near conspecific eggs on the leaf surface could lead to a dilution effect [15], with decreased predation risk compared to laying eggs on the leaf surface alone. Offspring laid on the leaf surface can feed right after hatching, whereas those on webs first need to move onto the leaf surface before feeding. Thus, although oviposition on the web is beneficial in terms of predation avoidance [13,14], it may bring costs for nymph growth. We suggest that *T. kanzawai* females choose oviposition site corresponding to the current presence of conspecific eggs, and that their choice may promote effective predation avoidance.

Considering the result in Exp. 1, we propose that experience of cohabitation with conspecific eggs for 19 h is likely to induce threat− females to oviposit on the leaf surface in the current absence of conspecifics. In contrast, threat-experienced *T. kanzawai* females laid more eggs on the web even in the absence of current threat over a prolonged period (figure 4*a*). Oku *et al*. showed that *T. kanzawai* females lay fewer eggs in the presence of the predatory mite *Neoseiulus womersleyi* [26]. The total number of eggs/female/day on day 1 was not significantly different between threat+ and threat− females (figure 4*b*). This suggests that threat-experienced mites indeed perceived no predator on the new leaf. As mentioned above, the web is a safer site than the leaf surface for eggs [13,14], and in fact eggs laid on the web are less frequently predated in an open environment where the predatory mite *N. womersleyi* can disperse easily [14]. These results suggest that *T. kanzawai* females appear to change oviposition site in a new situation based on threat experience, functionally preparing against future egg predation.

On the other hand, the oviposition site shift in threat+ females was not permanent, as shown by the gradually decreasing proportion of eggs laid on the web across 4 days (figure 4*a*). The reason for this decrease is that the number of eggs laid on the leaf surface increased over time (figure 4*c*). As *T. kanzawai* females on the web cannot directly access the leaf surface for feeding, they may be unlikely to shift completely from leaf surface to web. Because threat+ females also cohabited with eggs (figure 2*b*), they should have detected both conspecific eggs and predation risk. The threat-experienced females may have given more weight to predation risk than benefits of conspecific eggs laid together, and persisted to oviposit on the webs even after they were in a safe environment. As shown in the number of eggs/female/day along with the number of eggs/female/4 days, threat-experienced *T. kanzawai* females tended to lay eggs hurriedly. This may suggest that oviposition rate is adjustable in response to experience within the putative constraint of lifetime fecundity.

In Exp. 3, *T. kanzawai* females cohabited with conspecific males laid a lower proportion of their eggs on the web compared with alone females (figure 5). Considering the procedure of Exp. 3 and the result of Exp. 2, all females in Exp. 3 must have been affected by predator experience, because the proportion of eggs laid on the web by ‘alone’ females was equivalent to those of threat− females in Exp. 2. Therefore, the presence of males affected the experience-induced oviposition site shift in *T. kanzawai* females. This result clearly demonstrates flexibility in this experience-induced behavioural change. In contrast, the presence of conspecific female deutonymphs did not affect the behaviour of *T. kanzawai* females. In the closely related species *T. urticae*, copulations 24 h after the first copulation do not result in fertilization [27], reflecting lower significance of males for mated females. Oku [28] suggested that males copulate even with 1-day-old mated females in the absence of virgin females, and mated *T. uricae* females in the presence of a male spent less time feeding or ovipositing due to disturbance by male, resulting in diminished egg production. Although the number of eggs/female/day was not affected by conspecifics in Exp. 3, it is assumed that females frequently mated with males during cohabitation. Cues from copulatory attempts by males might have been sufficient for *T. kanzawai* females to recognize the presence of conspecifics, compared with cues from deutonymphs who do not usually contact with conspecifics. For *T. kanzawai* females that usually live in a group [16], frequent encounters with group members imply the presence of conspecific eggs, which may have induced females' oviposition on the leaf surface.

In contrast to *T. kanzawai*, predator-experienced *T. urticae* females shifted their oviposition site only on day 1 (figure 6*a*), and the number of eggs laid on the web was significantly lower than on the leaf surface (figure 6*c*). The speed of oviposition did not change in this species in response to predator experience (figure 6*b*). These results suggest that *T. urticae* retains the experience-induced behavioural change for less time than *T. kanzawai*, and that predator experience has lesser effects on oviposition behaviour in the former. In this context it is of interest to compare the natural ecology of these two closely related species. As already mentioned, *T. kanzawai* can deform leaves of their host plant, which facilitates construction of three-dimensional webs [12]. Hence oviposition on the web is probably more practical for *T. kanzawai* than for *T. urticae*. Furthermore, *T. urticae* has developed physiological resistance against more than 100 pesticides [29], and lives in agro-ecosystems where predators are relatively scarce due to frequent pesticide applications [21]. Therefore, reduced retention of experience-induced oviposition on the web in *T. urticae* may have been favoured in ecological conditions where they have less need to prepare for repeated predator encounters.

## 5. Conclusion

We have shown the following: (i) *T. kanzawai* females oviposited on the leaf surface in the presence of conspecific eggs. (ii) After experiencing predation threat, *T. kanzawai* females changed oviposition sites on the web even in the absence of further immediate predation threat. (iii) The experience-induced oviposition site shift by *T. kanzawai* females was flexible and varied with the presence of conspecific males. (iv) The experience-induced oviposition site shift was more short-lived in *T. urticae* than in *T. kanzawai*, possibly due to differences in their natural ecology. These results strongly suggest oviposition changes in *T. kanzawai* are based on a learning system involving multiple stimuli from conspecifics and predators. The flexible change of oviposition site in *T. kanzawai* females appears to be regulated by those stimuli perceived both in the past and at present. Thus, we can assume that a complex learning system more than a simple sensitization has been developed in *T. kanzawai*. It is especially noteworthy that spider mites, species with an extremely simple nervous system [30], show such multi-regulated and flexible experience-induced behaviour.

## Supplementary Material

ESM1 Dataset

## Supplementary Material

ESM2 Dataset

## Supplementary Material

ESM3 Dataset

## Supplementary Material

ESM4 Dataset

## Data accessibility

All data tables are available as the electronic supplementary material, S1 to S4. Electronic supplementary material 1 Dataset: The number of eggs laid on the web or on the leaf surface by each replicate in Exp. 1. Electronic supplementary material 2 Dataset: The number of eggs laid on the web or on the leaf surface by each replicate in Exp. 2. Electronic supplementary material 3 Dataset: The number of eggs laid on the web or on the leaf surface by each replicate in Exp. 3. Electronic supplementary material 4 Dataset: The number of eggs laid on the web or on the leaf surface by each replicate in Exp. 4.
